# Salt stress influences the proliferation of *Fusarium solani* and enhances the severity of wilt disease in potato

**DOI:** 10.1016/j.heliyon.2024.e26718

**Published:** 2024-02-20

**Authors:** Rahul Kumar Tiwari, Milan Kumar Lal, Ravinder Kumar, Vikas Mangal, Awadhesh Kumar, Rakesh Kumar, Sanjeev Sharma, Vinay Sagar, Brajesh Singh

**Affiliations:** a Division of Plant Protection, ICAR-Central Potato Research Institute, Shimla, HP 171001, India; b Division of CPB&PHT, ICAR-Central Potato Research Institute, Shimla, HP 171001, India; c Division of Crop Improvement, ICAR-Central Potato Research Institute, Shimla, HP 171001, India; d Division of Crop Physiology and Biochemistry, ICAR-National Rice Research Institute, Cuttack 753006, Odisha, India; e Division of Plant Pathology, ICAR-Indian Agricultural Research Institute, New Delhi 110012, India; f Division of Crop Protection, ICAR-Indian Institute of Sugarcane Research, Lucknow, Uttar Pradesh 226002, India

**Keywords:** Salinity, Enzymatic activity, *Fusarium*, Virulence, Pathophysiology, *Solanum*

## Abstract

Soil salinity has emerged as a critical abiotic stress in potato production, whereas wilt disease, caused by *Fusarium solani,* is the significant biotic stress. An experiment was performed to decipher the occurrence of wilt incidence by *F*. *solani* FJ1 under the influence of salinity in both *in vitro*and pot culture conditions. High salt concentration negatively influenced root and shoot development in the variety “Kufri Jyoti” but positively affected the mycelial growth and sporulation behaviours of *F*. *solani* FJ1. There was abundant whitish mycelial growth with enhanced biomass and high sporulation (microconidia production) in *F*. *solani* FJ1 cultured on salt-supplemented media. Moreover, under high salinity conditions (EC 2–8 dS m^−1^), severe wilting and rotting of vascular bundles were observed in plants artificially inoculated with *F*. *solani* FJ1. The mortality rate of potato plants was significantly higher under individual and combined stresses as compared to control. The wilt index of individual and combined stressed plants was also substantially higher compared to the control. Additionally, compared to the control, there was a significant decrease in total chlorophyll content and membrane stability index of the leaves under combined stress. However, the total phenols were increased under stress conditions. The total sugar content of potato plants decreased in infected plants, but increased when exposed to salt stress or a combination of salt stress and pathogen infection. *F*. *solani* infection also increased the activity of peroxidase (POX) and decreased the activity of phenylalanine ammonia-lyase (PAL) and catalase (CAT). These results suggest that *Fusarium* wilt and dry rot will be a more severe disease for potato cultivation in saline soils.

## 1 Introduction

Potato (*Solanum tuberosum* L.) is a widely consumed cash crop, with over one billion people in 150 countries relying on it for sustenance and employment [1,2]. China, India, and Russia are among the top three global potato producers, with 99, 43.8, and 31.1 million tons produced in 2020, respectively [3]. In 2022, India alone produced 53.5 million tons of potato. Increasing population and processing industries place a great deal of pressure on enhancing potato production while minimizing losses caused by biotic and abiotic stresses. Due to its vegetatively propagated nature, potatoes are extremely susceptible to soil-borne and tuber-borne pathogens [4,5]. The production of potatoes has been negatively affected globally by more than 40 soil-borne diseases that damage the tubers, which are the most economically significant part of the plant [[6], [4], [5], [7], [8], [9], [10], [11]]. In recent years, potato wilt disease has become a significant problem in potato cultivation [9,12,13]. Various *Fusarium* species, such as *F*. *oxysporum* f. sp. *tuberosi*, *F. solani* f. sp. *eumartii*, and *F. sambucinum*, cause wilt in potatoes. A potato plant's roots carry these pathogens to the stem's xylem vessels, where they move on to the plant's leaves. It is associated with necrosis, yellowing of the lower leaves, discoloration of the vascular tissues, stunted growth, wilting, and ultimately death of the plant. The pathogen initially causes *Fusarium* wilt in the standing crop, but severe issues arise in cold stores where the tubers are infected with *Fusarium*, leading to dry rot. Over 13 pathogenic species of *Fusarium* have been associated with this disease worldwide [14], with *F. sambucinum* being the most destructive species accountable for dry rot and wilt disease, as reported in potato-growing regions such as China, India, and Algeria [[15], [16], [17]]. In Iran, several *Fusarium* species have been identified as the causal fungi of potato wilt disease, with *F. sambucinum* and *F. solani* being the most predominant species [18]. In Tunisian potato cultivars, *F. solani, F. oxysporum* f. sp. *tuberosi, F. sambucinum and F. graminearum* have been observed to be more frequent [19,20]. A *Fusarium* wilt outbreak in Tunisia has been reported to destroy 30–50% of the potato crop and reduce tuber quality [20]. In Algeria, a study identified 13 distinct species under the *Fusarium* and *Neocosmospora* genera. Out of these, three species - *F. oxysporum*, *F. venenatum*, and *Neocosmospora solani* - were specifically found in plants exhibiting signs of *Fusarium* potato wilt [16]. Conversely, two species, *F. culmorum* and *N. tonkinensis*, along with an isolate of *Neocosmospora* sp., were solely discovered in tubers suffering from potato dry rot. The remaining species - including *F. redolens*, *F.* cf. *tricinctum*, *F. sambucinum*, *F.* cf. *incarnatum*-*equiseti*, *F. nygamai*, *F. brachygibbosum*, and *N. falciformis* - were linked with both types of samples. Wilt and dry rot is commonly caused by *F. coeruleum* in Great Britain (Libert) [21]. *F*. *graminearum*, a common cereal fungus, has also been frequently reported as a cause of wilt and dry rot in potato crop in North Dakota and Michigan. In cold stores with rot-affected tubers, *F. sambucinum, F. oxysporum,* and *F. solani* are the most frequently intercepted species [10,22].

In farming areas and regions with irrigation systems, soil salinity is a widespread problem [23]. It poses a serious risk to crop output in the future as approximately, 1128 million hectares of global land is affected by soil salinity and sodicity [24,25]. Salt tolerance of potato crops is moderate, as it has a threshold for soil salinity (ECe) at 1.7 dSm^−1^ and irrigation water salinity (ECw) at 1.1 dSm^−1^. Potato species tolerant to low electrical conductivity (EC) of irrigation water suffer approximately a 50% yield reduction. However, for sensitive potato species, the yield reduction is lower at around 25% [24].

In contrast to other abiotic challenges, salinity lasts the whole plant life cycle, increasing the possibility that it may co-occur with biotic stresses during a crop season [26]. Different crops and pathogens respond differently to soil salinity in terms of disease development. Salinity stress significantly increases plants' vulnerability to soil-borne infections, as per previous reports [[27], [28], [29]]. In addition to common reactions, plant responses to combined stresses have distinctive metabolic and defense-related shifts [30,31]. Reactive oxygen species (ROS) are produced as a stress defense mechanism, leading to signal transduction and metabolic reprogramming as key responses [32]. Multiple elements, including MAPKs, transcription factors, enzymes, antioxidants and heat-shock proteins are involved in this reaction [26]. Superoxide dismutase (SOD), peroxidase (POX), and catalase (CAT) activities, among other antioxidant enzymes, are vital for increasing salt tolerance in many plants [6,33].

Due to simultaneous biotic and abiotic challenges, crop stress intensifies [23,[34], [35], [36], [37], [38]]. Climate change increases temperature fluctuations, droughts, and salinity stresses, which affect plant growth and yield [34]. At the same time, multi-resistant pathogens and pests are posing significant biotic threats. These intertwined stresses can compound plant response mechanisms, making a single stress management approach insufficient [34,[39], [40], [41], [42], [43]]. Drought, for example, can make plants more vulnerable to pests, while high temperatures can make them more contagious. Changing agricultural practices and globalization result in identifying new stress combinations, which emphasize the need for integrated crop management. Multi-stress tolerance breeding, precision agriculture, and harnessing the synergistic effects of beneficial microorganisms are becoming increasingly popular. Combinations of biotechnological, agronomic, and molecular tools can enhance plant resilience [38,41]. Genomic studies can identify stress-responsive genes, while bioinformatics tools can help understand complex stress interactions. Global challenges are intensifying, and a significant amount of research is being conducted in this area, emphasizing the need to address combined stresses holistically to ensure sustainable farming.

In potatoes and other solanaceous vegetables, however, no unique or conclusive antioxidative system profile has been observed when disease and salinity are combined. Thus, this study aimed to understand how potato plants change morpho-physiologically when they simultaneously undergo salinity stress and *Fusarium* infection. Furthermore, this study aimed to analyze fungal behaviour under salt stress conditions for pathogenicity and proliferation. It sheds light on how soil salinity affects potato plant wilt incidence and extent. Various plant and pathogen characteristics were examined, such as vascular bundle rot, mycelial growth, sporulation, and growth related parameters. Investigations on biochemical parameters such as chlorophyll content, membrane stability index, osmotic potential, total phenols, and sugar content provide insights into the biochemical responses of potato plants under combined stress conditions, which can contribute to the development of stress-tolerant potato varieties and the formulation of appropriate management practices.

## 2 Material and methods

### 2.1 Pathogen isolation and morphological identification

Plants micropropagated from the variety “Kufri Jyoti” were obtained from the ICAR-Central Potato Research Institute, Shimla, India. We ensured that the tubes used in all of these experiments were kept fresh, that the plants were uniform in size, and that they contained no contaminant fungal spores or dust particles. We isolated the pathogen from dry rot-infected tuber samples collected in Punjab and Uttar Pradesh under the present experiment. After excising the diseased and healthy portions from the infected tubers, the surface sterilization process was performed with 1% sodium hypochlorite for 1 min, 70% ethanol for 30 s, and washed with distilled water. These tuber fragments were kept on potato dextrose agar media (PDA) (Himedia, Bioscience, India) and incubated at 18 to 24 °C. Single spore isolation was performed to establish the pure culture of the isolated fungi. Fully developed pure fungal cultures were examined for several features, including colony appearance, the presence of aerial mycelium, the characteristics of macroconidia and microconidia, conidia length (L) and width (L) index (N = 100), and the morphology of chlamydospores. We examined these morphological characteristics with previously released information on *Fusarium* species to confirm our findings [44]. Furthermore, Koch's postulates were also confirmed by the pathogenicity tests of all the isolates on plants and tubers of the variety Kufri Jyoti. The spore concentration was adjusted to 2 × 10^6^ using the serial dilution method and spore counting with a hemocytometer. The mass multiplication of the pure culture in potato dextrose broth (PDB) was performed after confirming morphology and pathogenicity (Himedia, biosciences, India). A mycelial disc (6 × 6 mm) was taken from fully grown pure culture plates and kept on PDB at 18–24 °C for 5 days.

### 2.2 Molecular identification of pathogen

To extract genomic DNA through CTAB-mediated methods, the resulting mycelial balls were filtered, dried, and subsequently used for genomic DNA extraction [45]. Nanodrop and agarose gels were used to determine the quality of the DNA. After obtaining the purified DNA, a PCR reaction was prepared. For the 20 μL PCR reaction setup, 10 μL of Taq buffer was used along with 1 μL of forward and reverse primers and 2 μL of template DNA (at a concentration of 50 ng μL^−1^). The final volume was adjusted to 20 μL with 6 μL of nuclease-free water. The primers used for amplification of the purified DNA were specific to the fungus and targeted the translation elongation factor 1 alpha gene (EF1Fα- ATGGGTAAGGAAGACAAGAC and EF1Rα GGAAGTACCAGTGATCATGTT). As part of the PCR reaction, an initial denaturation step of 4 min at 95 °C was followed by 33 cycles at 95 °C for 30 s, 55 °C for 1 min, and 72 °C for 1 min. The final extension step was performed for 10 min at 72 °C. Using a 1.0% agarose gel and TBE running buffer, gel electrophoresis was conducted to visualize the amplicon. Ethidium bromide was used to stain the gel, and gel images were captured using a gel documentation system (INTAS, Germany). The visible amplicons under UV light were carefully removed from the gel using a gel extraction method according to the manufacturer's protocol, utilizing a Qiagen kit. The quality of the extracted DNA was subsequently assessed using both a Nanodrop spectrophotometer and by running a gel on agarose. Two selected isolates were then sent to Eurofins Genomics India Pvt. Ltd for direct sequencing.

### 2.3 Fungal proliferation pattern under salt stress

By culturing the isolate (FJ1) on PDA supplemented with varying salt concentrations, we assessed the radial growth of fungal colonies. The final salt concentrations were 0, 2, 4, 6, 8, and 10 dS m^−1^. In each plate, a 5 mm disc of active mycelia was inoculated at the center, and the radial growth was measured after seven days using a standard measuring scale. The plates were kept at 24 °C. Five plates per concentration were used for this assay. To assess the sporulation level of FJ1 at different salt concentrations, a haemocytometer was used under a light microscope to observe both micro- and macroconidia. From a seven-day-old FJ1 culture on PDA, a 5 mm diameter mycelial disc was obtained. A microcentrifuge tube containing 2 ml of distilled water was vortexed to release the mycelial disc spores and a drop of the suspension was placed on a haemocytometer. Under a light microscope, the spores were counted and the macroconidial percentage was determined for all the treatments. To assess spore germination percentage, pitted slides were used to place suspensions of spores. For the preparation of the spore suspensions, FJ1 was grown in 100 ml of PDB with varying salt concentrations in 250 ml conical flasks at 24 °C for 10 days. After harvesting the mycelial mat, the fresh weight was measured by drying it with filter paper towels, and the dry weight was measured after two days of oven drying at 70 °C.

### 2.4 Combined stress of soil salinity and *F. solani*

For *in vivo* trials, plastic pots with dimensions of 15 cm in diameter and 15 cm in length were utilized and filled with autoclaved soil as substrate. Salts were added to make the soil saline before transplanting the plants. The amount of salt to be added was determined using the Sodium Absorption Ratio (SAR). To create the saline soil, 10.6 L of double distilled water containing the calculated amount of salts was added. This resulted in a range of electrical conductivity (EC) in the soil varying from 2 to 10 dS m^−1^. In the next step, the soil was artificially infected with *F*. *solani* by pouring 20 ml of conidia suspensions with a concentration of 2 × 10^6^. Twenty-day-old Kufri Jyoti seedlings were transplanted into the potted soil. Each pot contained three seedlings, and after successful establishment, two healthy seedlings were retained. Following treatments were used in the experiment: T1: Control (no FJ1 or elevated EC); T2: 2 dS m^−1^; T3: 4 dS m^−1^; T4: 6 dS m^−1^; T5: EC 8 dS m^−1^ T6: EC 10 dS m^−1^. Alongside T2-T6, the respective controls (without *Fusarium*) were also maintained. For each treatment, 15 plants were used, and each treatment was replicated three times. The pots were placed in a greenhouse with a temperature of 30 °C ± 3, a 12-h photoperiod, and a relative humidity of 70%. Over 90 days, the plants were regularly observed for symptoms of *Fusarium* wilt disease. The disease index was recorded by the evaluation of the percentage of withered leaves on inoculated plants (number of diseased leaves divided by the number of all leaves) [16].

### 2.5 Physiological assays

The total chlorophyll and carotenoid content of the whole leaf samples after 30 days of inoculation were measured following the method described by Lichtenthaler and Wellburn 1983 [46]. The total sugar content in the samples was estimated using the method developed by Murniece et al., 2010 [47]. The absorbance of the sample at 620 nm was measured to determine the sugar content. The MSI of the leaf samples was assessed based on the method outlined by Saneoka et al., 2004 [48]. This index indicates membrane integrity under stress conditions.

### 2.6 Plant growth parameters

After 30 days of pathogen inoculation, various growth attributes of the plants and tubers were assessed. The plant length (root and shoot) and weight (root and shoot) were recorded, indicating the extent of development and overall biomass accumulation. To determine the dry weight of the roots and shoots, the respective samples were dried in an oven at 80 °C for 24 h. This process eliminated moisture content and allowed for an accurate measurement of biomass accumulation in the form of dry weight.

### 2.7 Antioxidant enzyme activity

The following assays were performed to assess the activity of antioxidant enzymes. Peroxidase (POX) activity: The leaves (0.5 g) were blended with 10 ml of sodium phosphate buffer (pH 6.5). This suspension was centrifuged, and the enzyme extract was obtained. POX activity was determined by measuring purpurogallin formation from pyrogallol. Incubation at 25 °C for 5 min was carried out with enzyme extract, phosphate buffer, pyrogallol, and hydrogen peroxide (H2O2). The reaction was stopped by adding sulfuric acid (H2SO4), and the absorbance was measured at 420 nm. Catalase (CAT) activity: The enzyme extract was mixed with hydrogen peroxide (H2O2) and phosphate buffer (pH 6.8). After incubation at 20 °C, sulfuric acid (H2SO4) was added, and the mixture was titrated against potassium permanganate (KMnO4) solution. CAT activity indicates the decomposition of hydrogen peroxide by the enzyme. Phenylalanine ammonia lyase (PAL) activity: Enzyme extract was mixed with sodium borate buffer (pH 8.8) and phenylalanine. After incubation, the reaction was stopped, and the conversion of l-phenylalanine to transcinnamic acid was measured at 290 nm. Polyphenol oxidase (PPO) activity: The assay was performed using a mixture of enzyme extract, sodium phosphate buffer, and catechol. The change in absorbance at 495 nm was monitored at 30-s intervals for 3 min. PPO activity indicates the oxidation of catechol by the enzyme.

### 2.8 Statistical analysis

Each parameter evaluated in this study was analysed in triplicate using SAS (version 9.0, SAS Institute Inc.) and Prism 6.01 (GraphPad Software Inc., San Diego, CA, USA). The significant differences between the treatments were analysed using a completely randomized design with a two-factor ANOVA. The mean values obtained from the treatment were compared with Tukey's multiple comparisons test at p ≤ 0.05 significance level.

## 3 Results

### 3.1 Morpho-molecular identification of fungal isolates

**Fig. 1:**
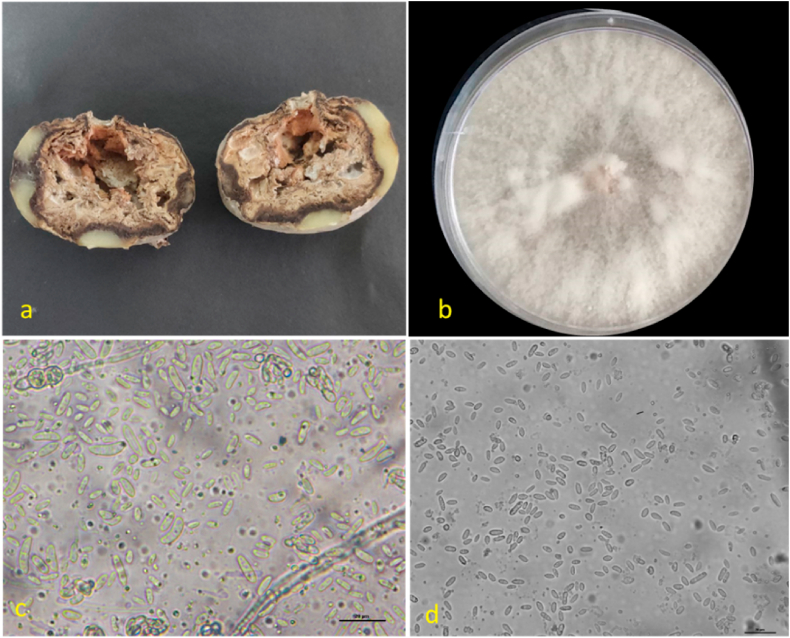
The colonies morphology of *F. solani* a) Infected sample b) Floccose white mycelial growth; c) Macroconidia d) Microconidia at 40× magnification (Scale Bar 20 μm).

The cause of potato wilt in the samples collected from Punjab and Uttar Pradesh, India, was determined through a combination of morphological and molecular identification techniques. The main objective was to identify the specific fungus species responsible for the development of the disease. The observed isolates displayed a culture morphology that was predominantly white to cream, with limited mycelium growth (Fig. 1a and b). Abundant cream-colored sporodochia were often produced. Conidiophores were present either singly or in small false heads, and macroconidia (L × B index 36.3 ± 4 × 3.8 ± 0.7) exhibited stout shape with a tapering apex (Fig. 1c). Microconidia (L × B index 13.5 ± 1.8 × 4.0 ± 0.3) were oval-shaped and typically had either no septa or a single septum, while chlamydospores were found either individually or in intercalary positions (Fig. 1d). A slight tan-brown discoloration was observed when these isolates were used to infect dry rot-affected tubers. The morphological characteristics observed in this study closely resembled those described for *F*. *solani*, as outlined in the *Fusarium* Laboratory Reference Manual [44].

Molecular studies were also conducted to supplement the morphological identification of *Fusarium* isolates. The TEF 1 sequence of isolates (FJ1and FJ2) matched 99–100 % with *F*. *solani* strain A8r4 (MK560287.1) and *F*. *solani* strain Zb-RR (MZ419443.1). These isolates were identified as *F. solani* FJ1 and *F*. *solani* FJ2, and their respective sequences have been submitted to GenBank under accession numbers OM319372 and OM3193. Likewise, the respective GenBank accession numbers of ITS sequences were OM190511 and OM190512.

### 3.2 Growth response of *F. solani* to saline solution

**Fig. 2:**
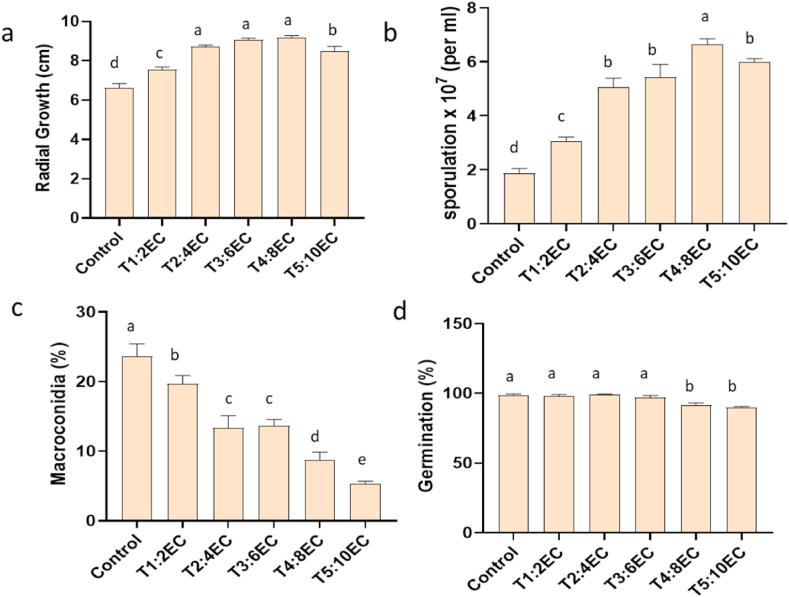
Mycelial growth and sporulation in *Fusarium solani* FJ1 at different salt concentrations. Radial growth (a), sporulation (b), macroconidia percentage (c) and spore germination (d). T1: 2 dS m^−1^; T2: 4 dS m^−1^; T3: 6 dS m^−1^; T4: EC 8 dS m^−1^; T5: EC 10 dS m^−1^. Bars represented as mean values ± SEM of three replicates. Different letters above the bars indicate statistically significant difference at P < 0.05.

**Fig. 3:**
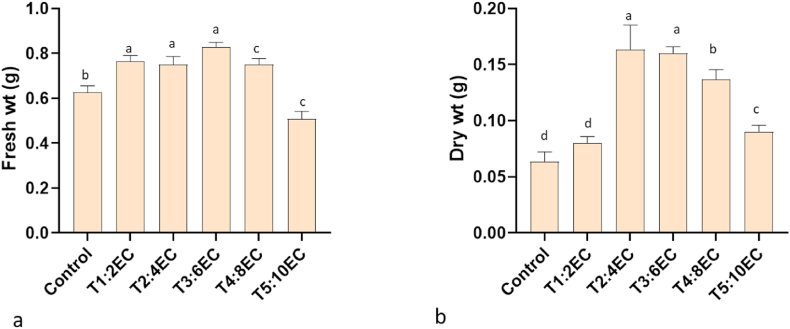
*Fusarium solani* FJ1 biomass accumulation at different salt concentrations. Fresh weight (a) and dry weight (b) on potato dextrose broth after ten days of inoculation. T1: 2 dS m^−1^; T2: 4 dS m^−1^; T3: 6 dS m^−1^; T4: EC 8 dS m^−1^; T5: EC 10 dS m^−1^. Bars represented as mean values ± SEM of three replicates. Different letters above the bars indicate statistically significant difference at P < 0.05.

There was a significant impact on fungal growth and proliferation under salinity stress, as observed by radial growth, germination percentage, sporulation behaviours and mycelial biomass. Periodic observation of fungal radial growth revealed a significant difference in control and salt-treated culture plates (P < 0.05). With increased EC levels (2–8 dS m^−1^), the fungus spread in terms of diameter was higher as compared to control (Fig. 2a). However, differences were non-significant at 4–8 dS m^−1^. Interestingly the sporulation of fungi varied significantly among all the treatments (P < 0.05). With increased EC levels (2–10 dS m^−1^), the sporulation almost doubled in the culture media (Fig. 2b). Enormous spores with abundant cream-colored sporodochia were observed in salt-treated media. Surprisingly, among these spores, the percentage of macroconidia was reduced at different salt concentrations (Fig. 2c). There was a significant reduction in the macrocondia at all treatment combinations as compared to control. However, increased salt concentration in the culture did not affect the germination percentages of the spore. There was a non-significant difference in germination percentage in control and 2–6 dS m^−1^ treatment combinations (Fig. 2d). However, a slight decrease in germination was observed at EC 8–10 dS m^−1^. The mycelial fresh weight and dry weight varied significantly among control and treatment (EC 8–10 dS m^−1^). Both fresh and dry weight increased significantly under salt stress (EC 2–6 dS m^−1^). Thereafter, the weights were significantly reduced (Fig. 3a and b).

### 3.3 Disease progression under stress conditions

**Fig. 4:**
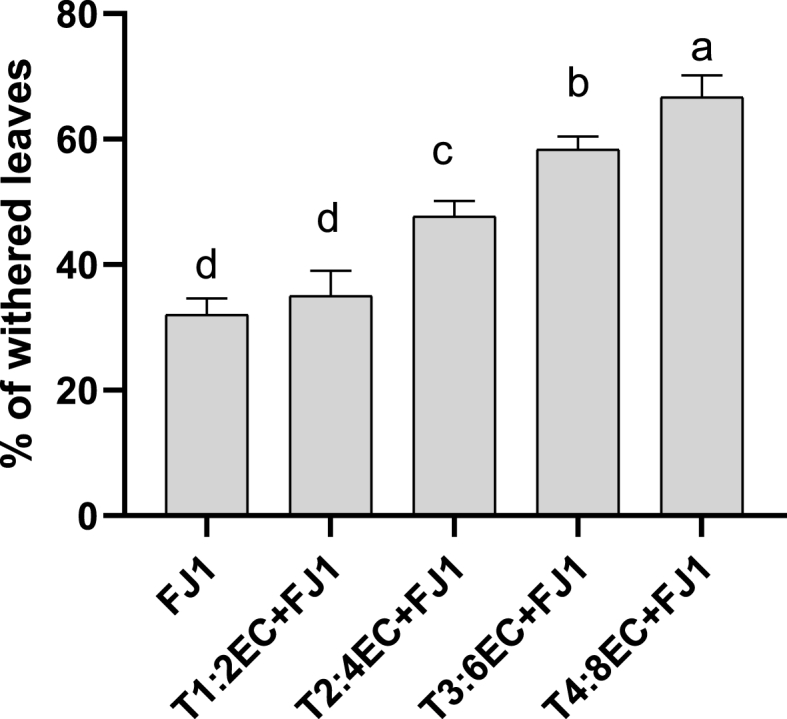
Wilt index (percent of withered leaves) in *Fusarium solani* FJ1 infected plants grown at different salt concentrations. Bars represented as mean values ± SEM of three replicates. Different letters above the bars indicate statistically significant difference at P < 0.05.

**Fig. 5:**
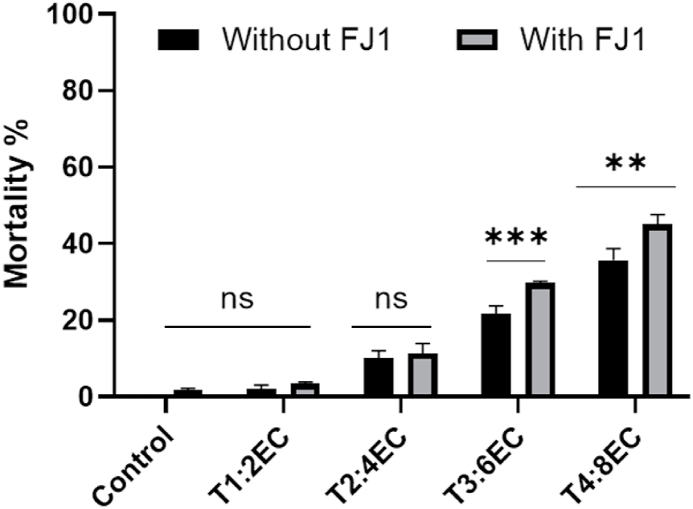
Mortality percentage in *Fusarium solani* FJ1 inoculated plants grown at different salt concentrations. Bars represented as mean values ± SEM of three replicates. * & *** indicates that mean values are significantly different (P < 0.05 & P < 0.001).

Severe root drying and discoloration of vascular bundles were observed in infected plants grown under salt stress compared to control plants. As per the disease index record, all the plants grown in EC 2–8 dS m^−1^ showed withered leaves. The percentage of withered leaves per plant (showing symptoms of hemiplegia or withered leaves) was in the range of 29–33% in the plants inoculated with fungus alone. However, under combined stress, the percentage of withered leaves was significantly higher at EC 4–8 dS m^−1^. Up to 60% of leaves were observed to be withered in infected plants grown under EC 8 dS m^−1^ (Fig. 4). Moreover, the mortality percentage was recorded higher in *Fusarium* infected plants grown at EC 6–8 dS m^−1^ (Fig. 5). The plants under combined stress depicted slowed growth, drooping of leaves and tender stems, coupled with an upward progression of symptoms, such as a gradual yellowing of leaves from the bottom to the top, leading to plant wilt and toppling. Control plants that were not inoculated remained symptom-free throughout the study.

### 3.4 Plant growth response

**Fig. 6:**
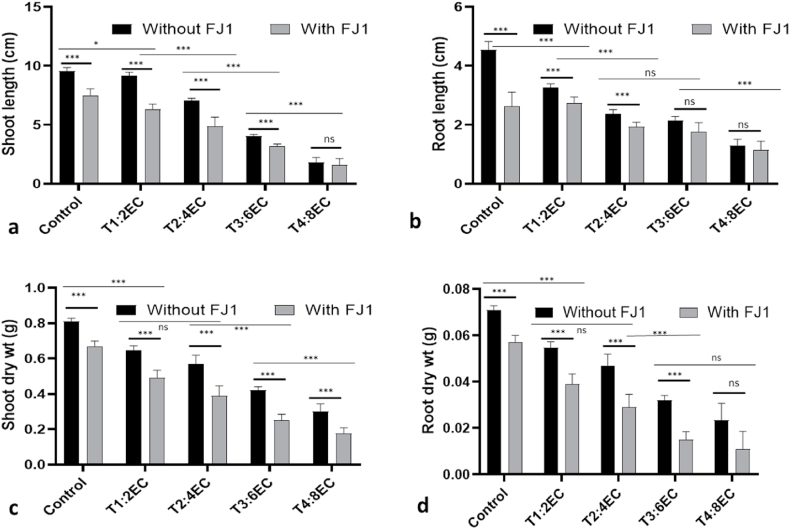
Plant growth parameters under *Fusarium solani* FJ1 infection at different salt concentrations. Shoot Length (a), Root Length (b), Shoot Dry Weight (c) and Root Dry Weight (d). T1: 2 dS m^−1^; T2: 4 dS m^−1^; T3: 6 dS m^−1^; T4: EC 8 dS m^−1^. Bars represented as mean values ± SEM of three replicates. * & *** indicates that mean values are significantly different (P < 0.05 & P < 0.001).

Individually, fungal infection and salt stress caused a significant reduction in plant growth parameters (shoot and root length and respective dry weights). However, the co-occurrence of both stresses caused a more detrimental effect on growth parameters. Root and shoot length were severely reduced in fungus-infected plants grown at EC 2–8 dS m^−1^. The percent reduction in shoot length was 36% and 78% at EC 4 dS m^−1^ and EC 8 dS m^−1^, respectively in *Fusarium* infected plants of Kufri Jyoti (Fig. 6a and b). Likewise, the root length was severely hampered up to 50% and 67% at EC 4 dS m^−1^ and EC 8 dS m^−1^, respectively. Overall, shoot and root dry weights also showed a similar pattern of consistent reduction under combined stress conditions (Fig. 6 c, d). A maximum reduction in shoot and root dry weight up to 73% and 76% was observed at EC 8 dS m^−1^, respectively.

### 3.5 Physiological parameters under combined stress

The effect of *F*. *solani* FJ1 and different salinity levels on the physiological parameters of potato (total chlorophyll, total phenolics, membrane stability index and total sugar) revealed a substantial decline in the overall health status of the plants. The chlorophyll content has deteriorated under the influence of fungal infection as well as under salinity stress. However, the combined stress caused a greater reduction in total chlorophyll content as compared to individual stress (Table 1). The percent reduction of chlorophyll content was in the range of 5–18.9 % in response to either salinity levels (2–8 dS m^−1^) or the combined stress factors. Similarly, the membrane stability index was substantially reduced in individual or combined stress conditions. At different salinity levels, the percent reduction was in the range of 33.7–64.9% while under co-occurrence of *Fusarium* infection, the percent reduction ranged from 28.5 to 64.9%. Tukey's multiple comparison analysis showed significant differences among treatments. The total sugar content and total phenolics were significantly enhanced by salinity stress, and the level of those attributes was significantly increased by simultaneous fungal infection. In response to increasing soil salinity, this increase became less apparent. The highest sugar content was observed in combined stress (FJ1+2 dS m^−1^) followed by FJ1+4 dS m^−1^ and FJ1+8 dS m^−1^. The total phenol content increased by 26.6%, and 15% at 2 and 4 dS m^−1^ but remained unaffected or decreased after subsequent increases in salinity levels. Under combined stress treatment FJ1 + 2 dS m^−1^, the percentage increase was 30% followed by subsequent reductions or non-significant differences at higher salinity levels.

**Table 1:** Effect of *Fusarium solani* FJ1 and different salinity levels on physiological parameters of potato.

Treatments	Total Chlorophyll (mg/g)	Membrane stability index (%)	Total Phenolics (mg/g)	Total Sugar Content (mg/g)
Negative control	1.79^a^	77^a^	6^b^	1.94^e^
Positive Control (FJ1)	1.68^b^	61^b^	5^c^	2.67^d^
2 ds/m	1.74^a^	51^c^	7.6^a^	1.87^e^
4 ds/m	1.69^b^	43^d^	6.9^a^	2.29^d^
6 ds/m	1.56^c^	35^e^	6.1^b^	2.67^d^
8 ds/m	1.55^c^	27^f^	5.8^b^	3.08^c^
2 ds/m + FJ1	1.71^a^	55^c^	6.5^b^	5.4^a^
4 ds/m + FJ1	1.64^b^	50^c^	5.9^b^	4.3^b^
6 ds/m + FJ1	1.45^d^	30^g^	4.6^c^	4.1^b^
8 ds/m + FJ1	1.50^b^	27^f^	4.1^c^	3.3^c^

Note: Different letters above the data indicate statistically significant difference at P < 0.05.

### 3.6 Antioxidant enzymes under *F. solani* infection and salinity stress conditions

**Fig. 7:**
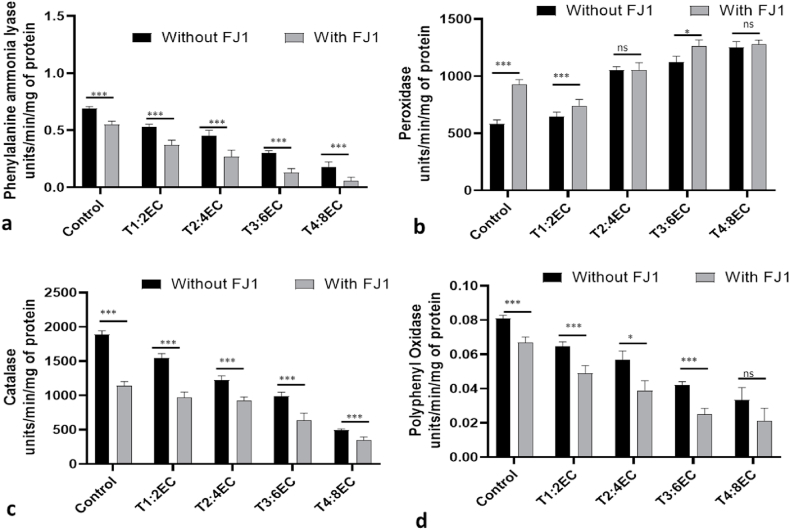
The activity of antioxidant enzymes under *Fusarium solani FJ1* infection at different salt concentrations. Phenyl ammonia lyase (a), Peroxidase (b), Catalase (c), Polyphenol oxidase (d). T1: 2 dS m^−1^; T2: 4 dS m^−1^; T3: 6 dS m^−1^; T4: EC 8 dS m^−1^. Bars represented as mean values ± SEM of three replicates. * & *** indicates that mean values are significantly different (P < 0.05 & P < 0.001).

The activity of antioxidant enzymes like PPO, PAL, CAT and peroxidase was significantly altered under individual or combined stress conditions. There was a significant reduction in PAL activity of control plants as compared to plants either grown in saline conditions or infected with *Fusarium.* The activity of this enzyme is further reduced under combined infection. In plants grown under different EC (2–8 dS m^−1^) the percent reduction was in the range of 28.5%–64.2% as compared to control. Likewise, In *Fusarium*-infected plants grown under different salinity levels*,* the percent reduction in PAL activity was in the range of 24%–80% (Fig. 7a). Catalase activity of likewise reduced in all the plants grown under stress conditions (individual or combined) as compared to control. The reduction ranged from 16.6% to 72.2% for salinity stress and 20.8%–79.1% for plants grown under combined stress conditions. Contrastingly the peroxidase activity was increased with an increase in salinity levels and Fusarium infection further enhanced the activity of peroxidase (Fig. 7b and c). However, as PAL and CAT the activity of PPO has significantly deteriorated in salinity stressed plants and *Fusarium* infected plants (Fig. 7d). The reduction in PPO levels was in the range of 25%–62.5% for salinity stress grown plants and 28.5%–57.1% in plants cultivated under combined stress conditions.

## 4 Discussion

More than 13 different *Fusarium* fungus species have been identified worldwide, and they all contribute to the wilt disease that affects potatoes [10,14]. *F. solani*, *F*. *sambucinum*, and *F*. *oxysporum* are the three most aggressive fungi that cause this disease in many regions of Europe, Africa, China, North America, and Asia. Previously, several reports highlighted the occurrence of *F*. *solani* as the main causal agent responsible for dry rot and wilt diseases in cultivated potatoes [[49], [50], [51]]. In this study, the *Fusarium* infected samples were collected from major potato growing regions of Punjab and Uttar Pradesh. The morphological and molecular identification established the identity of the causal agent as *F. solani.* The primers ITS and TEF 1 α have been extensively used in morpho-molecular identification of *Fusarium* species [10,52,53]. The infection of *F. solani* critically affects plant growth related parameters such as shot and shoot length, dry weight and other physiological parameters such as chlorophyll content, total phenols and total sugars [54]. A recent report highlighted the significant deterioration of nutritional quality parameters such as starch content, total soluble sugars, amylose content and other antioxidant metabolites in *F*. *solani* infected potato tubers of variety Kufri Jyoti and Frysona [22]. This study also revealed the impact of an abiotic stress, specifically salinity, on the behaviour of *F. solani* and its effects on various morpho-physiological parameters. The findings revealed significant alterations in these parameters under diseased conditions. However, the primary objective of the study was to understand how *F*. *solani* responds to salinity, which is a persistent stress factor throughout the plant's life cycle.

Soil salinity has a severe impact on agricultural production, and it particularly reduces the productivity of solanaceous crops such as potato [33,55]. The current study also revealed that salinity harms plant height, plant growth, and biomass development in potato. As the concentrations of NaCl in the soil increased, the negative effects became more pronounced. Mainly the EC 4–10 dS m^−1^ proved detrimental to plant growth. The effect of soil salinity on disease development varies across different crops and pathogens. The research findings indicate that salinity stress significantly increases the susceptibility of plants to soil-borne pathogens [56,57]. Laboratory experiments were conducted to assess the proliferation and pathogenicity of *F*. *solani* FJ1 at different levels of EC (EC 2–10 dS m^−1^). The results indicated that fungal radial growth, sporulation and germination percentage were increased significantly at EC range of 2–4 dS m^−1^. It was previously observed that *F*. *oxysporum* f. sp. *ciceris* mycelial growth rate and biomass of was also significantly affected in high NaCl concentrations. As the concentration of NaCl was increased up to 400 mM, the rate of mycelial growth and biomass increased, and then decreased as salt concentrations were increased up to 600 mM. It was found that mycelial growth rate and biomass increased with an increase in NaCl concentration up to 400 mM. As salt concentrations continued to increase (up to 600 mM), both mycelial growth rate and biomass decreased. However, beyond this concentration, further increase in salt concentrations (up to 600 mM) led to a decline in both mycelial growth rate and biomass. The mycelial fresh weight and dry weight exhibited significant variations between the control group and the treatment group subjected to electrical conductivity levels of 8–10 dS m⁻^1^. Under salt stress conditions (EC 2–6 dS m⁻^1^), both fresh weight and dry weight showed a notable increase. However, beyond this range, the weights experienced a significant reduction. According to the findings by Shoaib et al., 2018 the endurance of *F*. *oxysporum* f. sp. *cubense* at different electrical conductivity levels revealed that fungal biomass showed a significant increase of 50–90% within an EC range of 2–4 dS m⁻^1^ compared to the control. Furthermore, with a further increase in EC from 5 to 8 dS m⁻^1^, fungal biomass exhibited a 33% increase compared to the control.

The fungal infection in plants grown under electrical conductivity levels of 2–8 dS m⁻^1^ resulted in a significant reduction in both root and shoot length. In *Fusarium*-infected plants of the Kufri Jyoti variety, there was a 36% reduction in shoot length at EC 4 dS m⁻^1^ and a more severe reduction of 78% at EC 8 dS m⁻^1^. Overall, shoot and root dry weight also showed a similar pattern of consistent reduction under combined stress conditions. A maximum reduction in shoot and root dry weight up to 73% and 76% was observed at EC 8 dS m^−1^, respectively. Our findings were in consensus with the previous report, where a concentration of 200 mM NaCl had a significant negative impact on seed germination and caused a substantial reduction in chickpea biomass [26]. According to the findings reported by Shoaib et al., in 2018, the combination of *Fusarium* infection and salinity stress resulted in increased disease severity. This combined stress condition exhibited the highest disease incidence, with 63% of plants affected and a significant increase in plant mortality, reaching 50%. Furthermore, the bulb morphology was severely deteriorated, leading to the highest reduction in diameter (50%) and weight (∼80%) compared to plants experiencing either individual stress (*Fusarium* infection or salinity stress) alone. These results highlight the synergistic negative effects of simultaneous *Fusarium* infection and salinity stress on the overall crop health.

The study demonstrated that both fungal infection and salinity stress had a detrimental impact on chlorophyll content. However, the combined stress condition resulted in a greater reduction in total chlorophyll content compared to individual stress factors alone. The percent reduction in chlorophyll content ranged from 5% to 18.9% in response to different salinity levels (2–8 dS m⁻^1^) or the combined stress factors. These findings indicate that the simultaneous presence of fungal infection and salinity stress exacerbates the negative effect on chlorophyll content, leading to a more pronounced reduction compared to each stressor individually. Previously, a depreciation in the photosynthetic pigments content in paddy, wheat and *Pisum sativum* has been due to either salinity stress [[58], [59], [60]] or fungal infection [[61], [62], [63]]. Under combined infection of *Fusarium* and salinity, a reduction in chlorophyll content has been documented in chickpeas and onions.

The activity of antioxidant enzymes like PPO, PAL, CAT and peroxidase was significantly altered under individual or combined stress conditions. There was a significant reduction in PAL, CAT and PPO activity of control plants as compared to plants either grown in saline conditions or infected with *Fusarium.* Oxidative stress injury of cellular apparatus could be due to enhanced production of peroxidase, while decreased accumulation of CAT and PAL [64]. The activity of phenylalanine ammonia lyase (PAL) was observed to decrease in this study. This decrease in PAL activity may lead to an accumulation of certain phenolic components, such as cinnamic acid. The presence of elevated levels of cinnamic acid can inhibit flavonoid biosynthesis and further hinder the activity of PAL. Consequently, the reduced PAL activity can disrupt the biosynthesis of flavonoids and other related compounds [65]. The decreased catalase (CAT) activity observed in this study might be due to overproduction and accumulation of hydrogen peroxide (H_2_O_2_). This accumulation of H_2_O_2_ can induce oxidative stress within the cell. Additionally, oxidative stress can trigger the activation of peroxisomal endopeptidases, leading to enhanced proteolysis. The combined effects of increased H_2_O_2_ levels and enhanced proteolysis contribute to the oxidative damage and disruption of cellular processes associated with oxidative stress [66]. *Fusarium* infection in plants induces wilting symptoms through the systematic production of toxins, primarily Fusaric acid. This toxin has been found to decrease stem hydraulic conductance and leaf water potential. These changes result in membrane injury and lead to water leakage from damaged cells, as previously described by Van Alfen and Turner, 1975 [67]. Furthermore, Wang et al., 2015 observed that the reduction in net photosynthesis caused by *Fusarium* infection leads to stomatal closure and disrupts metabolic pathways involved in photosynthesis [68]. This disruption in photosynthetic processes has a negative impact on plant sugar accumulation and overall biomass production. These findings highlight the multifaceted effects of *Fusarium* infection and salinity on plant physiology and metabolism, ultimately leading to wilting symptoms and reduced plant performance.

## 5 Conclusion

This study investigated the effects of stress on wilt severity and virulence mechanisms of the fungal pathogen *F. solani* FJ1 in potato. The results showed that soil salinity had a favourable impact on the growth of FJ1, promoting mycelial growth and sporulation. Combined stress led to an early and severe wilt development in potato plants. Furthermore, the saline environment induced a shift in fungal proliferation and virulence. Under saline conditions, *F*. *solani* adopts a more aggressive and destructive mode of infection in the host plant. Understanding how soil salinity influences the growth and virulence of *F*. *solani* can help develop more effective disease management strategies. Farmers and researchers can focus on mitigating salinity stress in potato cultivation to reduce the severity of wilt and minimize crop losses. Identifying the specific mechanisms by which *F. solani* adapts and becomes more aggressive under saline conditions can guide the breeding of resistant potato cultivars. This information can be valuable for assessing the potential impact of soil salinity on crop diseases in regions affected by increasing salinization due to factors like climate change or poor irrigation practices. Overall, this study provides insights into the complex dynamics between stress factors, pathogen behavior, and disease development. These insights can inform future research and practical measures to mitigate the impact of *Fusarium* wilt in potato.

## Funding

This work was supported by Indian Council of Agricultural Research. (ICAR),India.

## Data availability statement

All data generated or analysed during this study are included in this published article.

## CRediT authorship contribution statement

**Rahul Kumar Tiwari:** Writing – original draft, Methodology, Conceptualization. **Milan Kumar Lal:** Writing – review & editing, Conceptualization. **Ravinder Kumar:** Formal analysis. **Vikas Mangal:** Investigation. **Awadhesh Kumar:** Resources. **Rakesh Kumar:** Methodology, Formal analysis. **Sanjeev Sharma:** Formal analysis. **Vinay Sagar:** Resources. **Brajesh Singh:** Supervision, Resources.

## Declaration of competing interest

The authors declare the following financial interests/personal relationships which may be considered as potential competing interests:Corresponding author is associate editor of this journal. If there are other authors, they declare that they have no known competing financial interests or personal relationships that could have appeared to influence the work reported in this paper.
